# The Use of Dietary Additives in Fish Stress Mitigation: Comparative Endocrine and Physiological Responses

**DOI:** 10.3389/fendo.2019.00447

**Published:** 2019-07-10

**Authors:** Marcelino Herrera, Juan Miguel Mancera, Benjamín Costas

**Affiliations:** ^1^IFAPA Centro Agua del Pino, Huelva, Spain; ^2^Department of Biology, Faculty of Marine and Environmental Sciences, Campus de Excelencia Internacional del Mar (CEI·MAR), Instituto Universitario de Investigación Marina (INMAR), Universidad de Cádiz, Cádiz, Spain; ^3^Centro Interdisciplinar de Investigação Marinha e Ambiental (CIIMAR), Matosinhos, Portugal; ^4^Instituto de Ciências Biomédicas Abel Salazar (ICBAS-UP), Universidade do Porto, Porto, Portugal

**Keywords:** fish, stress mitigation, additive, welfare, cortisol

## Abstract

In the last years, studies on stress attenuation in fish have progressively grown. This is mainly due to the interest of institutions, producers, aquarists and consumers in improving the welfare of farmed fish. In addition to the development of new technologies to improve environmental conditions of cultured fish, the inclusion of beneficial additives in the daily meal in order to mitigate the stress response to typical stressors (netting, overcrowding, handling, etc.) has been an important research topic. Fish are a highly diverse paraphyletic group (over 27,000 species) though teleost infraclass include around 96% of fish species. Since those species are distributed world-wide, a high number of different habitats and vital requirements exist, including a wide range of environmental conditions determining specifically the stress response. Although the generalized endocrine response to stress (based on the release of catecholamines and corticosteroids) is detectable and therefore provides essential information, a high diversity of physiological effects have been described depending on species. Moreover, recent omics techniques have provided a powerful tool for detecting specific differences regarding the stress response. For instance, for transcriptomic approaches, the gene expression of neuropeptides and other proteins acting as hormonal precursors during stress has been assessed in some fish species. The use of different additives in fish diets to mitigate stress responses has been deeply studied. Besides the species factor, the additive type also plays a pivotal role in the differentiation of the stress response. In the literature, several types of feed supplements in different species have been assayed, deriving in a series of physiological responses which have not focused exclusively on the stress system. Immunological, nutritional and metabolic changes have been reported in these experiments, always associated to endocrine processes. The biochemical nature and physiological functionality of those feed additives strongly affect the stress response and, in fact, these can act as neurotransmitters or hormone precursors, energy substrates, cofactors and other essential elements, implying multi-systematic and multi-organic responses. In this review, the different physiological responses among fish species fed stress-attenuating diets based on biomolecules and minerals have been assessed, focusing on the endocrine regulation and its physiological effects.

## Introduction

The study of stress in fish has significantly increased in the last years, mainly due to its close connection to animal welfare. It is widely accepted that a good fish welfare ensures a successful culture in fish farms, as in superior animal facilities. In this way, fish farmers are progressively recognizing it, since survival and growth, among other factors, are known to decrease under poor welfare conditions (1).

In spite of the negative perception of stress, it has been reported that, at low levels, it leads to a necessary and suitable response for adapting organisms to new environment/conditions; which is called eustress (2, 3). In contrast, distress is referred to a more severe and continuous stressful condition having suppressor effects on immune system and impairing physiological functions (4).

In fish farming, several zootechnical systems and variables are adjusted to achieve the maximum animal welfare without affecting the productive yield, though sometimes the right balance is very difficult to find. Besides the technological and infrastructural adaptations, the use of new feeding strategies is an easy and practical procedure to improve the fish welfare. In this context, the concept of functional food (providing beneficial effects on the organism besides the nutritional ones) has arisen as a new method to improve the general healthy status, including welfare (5). By this reason, several works on fish farming are based on the addition of specific substances with biological activity to conventional commercial fish feed in order to modulate or attenuate the stress response and, hence, improve the welfare (6–11). Those works focus on the stress response in fish fed experimental feeds, after submitting them to stressful procedures as netting, air exposure, high stocking density, chasing, and others. The diversity is very high, reporting many types of stressors and additives, and species, and, despite the methodological approach is similar, a wide range of stress markers (e.g., hormones, enzyme activity, immune parameters, gene expression, etc.) have been reported (12–15). The final goal is to find the most suitable additive and feeding strategy (i.e., time, concentration) to prevent fish from suffering, especially for typical stress-related processes in fish farming (e.g., grading, vaccination, fishing, etc.).

The stress response as a complementary study to nutritional issues has been carried out in many works, especially those on different protein, lipid, or carbohydrate concentrations and ratios in the diet (16–18). In this sense, those papers were, probably, the first evidences of dietary effects on the fish stress response (19). At the same time, vitamins (mainly ascorbic acid) were also target substances in that type of studies (20, 21). Lastly, thanks to new biotechnological protocols developing new substances, isolating/extracting biomolecules more efficiently, or including any additive in commercial feeds, many works have also described the effects of specific molecules (e.g., amino acids, nucleotides, polysaccharides, etc.) on the stress response (22–24).

**Figure 1:**
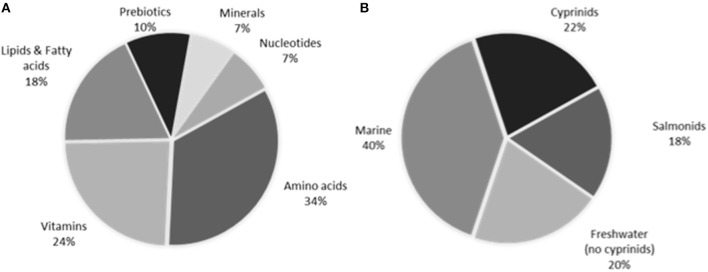
Percentage of works for **(A)** every additive type, and **(B)** every fish species group type in the literature dealing with stress attenuation through feed additives in fish.

For the last decades, studies dealing with proteins and amino acids have been the most abundant (Figure 1). The versatility of amino acids may justify their first place in this ranking, since some of them are directly involved in the neuroendocrine response. Fatty acids have also been frequently studied, especially those related to nutritional requirements (docosahexanoic, arachidonic, and eicosapentanoic acids). Some nucleotides, including trademarks, are progressively being assayed in fish; in spite of being stress alleviators, its interaction with the stress axis still remains unclear (25).

In this review, the literature on fish stress mitigation through feed additives based on biomolecules and minerals has been revised and analyzed, aiming at comparing the endocrine and physiological responses along farmed fish species.

## The Endocrine Stress Response in Fish

Stress responses have been deeply studied in fish, showing the key role of the endocrine system in the process. The primary stress response is based on hormonal cascades; in fact, the stress response was initially referred as the general adaptative syndrome (GAS), consisting of a hormonal cascade which promotes the other responses to stressors (3). HPI (i.e., hypothalamus-pituitary-interrenal) and HSC (i.e., hypothalamic-sympathetic-chromaffin) axes are activated during this primary response, releasing corticosteroids and catecholamines (e.g., adrenaline, nor-adrenaline and dopamine) into the blood stream. Following several energy metabolic pathways are enhanced (secondary response) and, if stress stands, severe failures at organism level (e.g., pathologies, decreasing growth, dead) may appear (tertiary response) (26).

The hormonal cascade starts at the hypothalamus level, which secretes the corticotropin releasing hormone (CRH) to stimulate the pituitary for releasing ACTH (i.e., adrenocorticotropic hormone) and MSH (i.e., melanophore stimulating hormone) into the blood stream. As a result, chromaffin cells, and interrenal cells from the head-kidney release catecholamines, and cortisol, respectively. Therefore, plasma cortisol and catecholamines are considered good acute stress markers. In fact, adrenaline is considered to be the stress hormone, and cortisol the adaptive hormone (27). The effects of cortisol on energy metabolism and other physiological functions is already known in fish, indeed it is the responsible of the releasing of energy substrates to the blood stream (secondary response), stimulating glycolysis, and other metabolic pathways (28). The catecholamines role in the stress metabolic response is poorly known in fish, meanwhile it is known that affect carbohydrate and lipids metabolism in mammals (29).

Thanks to the development of powerful tools on molecular biology, the knowledge of the HPI signaling in teleosts has progressed significantly. Many corticosteroid precursors and receptors have already been characterized in several species, providing valuable data in the field (30–32). Therefore, the classical stress markers (plasma hormones, immune parameters, metabolic rates) are currently studied together specific molecular biomarkers. Eissa and Huang (33) have revised thoroughly all genes involved in the fish stress response depending on stressor type, and stated that the use of genomic tools to study the candidate genes associated with stress responses are often unique signatures or imprints of specific stressors and could determine early signs of stressors. Having this in mind, Kiilerich et al. (34) have recently studied the expression of glucocorticoid and mineralocorticoid receptors (i.e., GR1, GR2, and MR) at different levels, concluding that the control and release of cortisol after stress is regulated through a negative cortisol feedback occurring at pituitary level; to the date, it was thought that this feedback occurred at every level of the HPI axis. Other authors have concluded that cortisol regulation is also dependent on circulating glucose concentration under acute stress, reporting a stimulatory effect of increasing glucose levels on the cortisol release (35). Despite the latest progress in the subject, the regulation of stress axis, and mechanisms of cortisol action in fish still remains unclear. In this sense, Faught et al. (36) suggested that future studies should be focused on the rapid non-genomic effects of cortisol, since that pathway could be crucial in the transcriptional activation of non-GR target genes during stress.

In the study of other endocrine factors and hormones, beyond the “classical” cortisol and catecholamines, involved in the fish stress response, the leptins have been objective for years (37–40). It seems clear that leptin interacts with the HPI axis at both head-kidney and pituitary gland levels, though contradictory results have been published on ACTH stimulation (37, 41). Gorissen and Flik (41) have stated that this hormone may convey information on energy status and serve to downplay the stress response, contributing to the coordination of the balance between eustress and distress.

Continuing on new hormones and endocrine responses, Skrzynska et al. (42) have recently studied the involvement of the vasotocinergic and isotocinergic systems in the stress response. These authors have stated that changes in *avt* (arginine vasotocin) and *it* (isotocin) gene expression, and in their specific receptors (*avtrv1, avtrv2*, and *itr*) at central (hypothalamus and pituitary) and peripheral (liver and head-kidney) locations, demonstrate that vasotocinergic and isotocinergic systems could have a role in several physiological changes induced by air exposure, including metabolic and energy repartitioning processes as well as the control of synthesis and release of several hormones as the final product of different endocrine pathways.

Lastly, a very innovative and recent study has revealed the cytoprotective importance of the CRH in the stress-induced apoptosis during the ontogeny (43). These authors have demonstrated the relation between CRH and caspase-3 activity (an effector caspase that execute apoptosis) during zebrafish (*Danio rerio*) ontogeny. They also highlighted that it can be a novel function for CRH during a period of embryonic development when the HPI axis is not yet matured, and proposed that it may help mediating the impacts of early life stress on offspring phenotype.

Summarizing, the literature on endocrine responses to stress in fish is extensive, and significant advances have been achieved for the last years. A consensus exist on the HPI (and HSC) response after stress and the roles of the main factors, including tissues where they act. Nevertheless, the interaction of the axis with other endocrine or metabolic processes is poorly understood. In most of cases, it has been stated that interaction exists (thanks to powerful bioindicators) though the intrinsic biochemical, physiological and endocrine processes involved in it have not been described yet.

## Physiological Roles of Dietary Additives

Additives are added in food to both improve the physiological effects on the consumer (probiotics, prebiotics, etc.) and provide/modify some physical food properties (texture, taste, color, etc.). The first group includes the stress attenuation, and diverse works on fish welfare have focused on it. The general biological functions and physiological roles of those additives on the fish stress response are summarized in Table 1. For the last 20 years, over 30 biomolecules and minerals, and around 38 fish species have been assayed in this subject. Below a more detailed revision depending on every additive group and it main physiological effects are shown.

**Table 1:** Main additives and its physiological effects assayed in fish in experiments based on the study and reduction of the stress response.

**Substance**	**General biological functions**	**Biological function related to stress system (described in fish)**
Amino acids^1^	Enzymes, antibodies, hormones, pH regulation, cell signaling, muscle structure	Neurotransmitter and hormone precursors, anti-oxidative enzymes, enhancer of fatty acid oxidation
Vitamins^2^	Enzyme cofactor, antioxidants	Enzyme cofactor, antioxidant, immunostimulant
Lipids and fatty acids^3^	Building biological membranes, storing energy	Energy reserves, eicosanoid precursors
Prebiotics^4^	Storing and providing energy, building macromoecules	Energy source, prebiotic
Nucleotides^5^	Nucleic acids building, cell signaling	Immune system enhancer
Minerals^6^	Bone and tooth building, energy production, muscle function, enzyme cofactor, antioxidant	Enzyme cofactor

1 *Morrow et al. (17), Hog¨lund et al. (44), Aragão et al. (45), Tejpal et al. (46), Abdel-Tawwab (47), Wolkers et al. (48), Conde-Sieira et al. (35), Hooley et al. (16), Kumar et al. (49), Morandini et al. (50), Chen et al. (51), Tian et al. (52), Liu et al. (24), Habte-Tsion et al. (14), Babaei et al. (12), Azeredo et al. (7), Herrera et al. (8), Cabanillas-Gámez et al. (6), Harpaz (53), Papoutsoglou et al. (54), Lepage et al. (55), Costas et al. (56), Costas et al. (57), Martins et al. (58), Hoseini et al. (59)*.

2 *Thompson et al. (60), Montero et al. (61), Chen et al. (62), Belo et al. (63), Trenzado et al. (64), Liu et al. (20), Liu et al. (13), Falahatkar et al. (65), Miao et al. (66), Guimarães et al. (67), Imanpoor et al. (21), Jia et al. (10), Cheng et al. (68), Jakab Sándor et al. (69), Alves Martins et al. (70), Hwang et al. (71), Davis et al. (72)*.

3 *Lochmann et al. (73), Van Anholt et al. (74), Van Anholt et al. (75), Bransden et al. (76), Alves Martins et al. (77), Trushenski et al. (78), Araújo and Rosa (79), Xu et al. (80), Rezek et al. (81), Martins et al. (82)*.

4 *Xie et al. (83), Torrecillas et al. (84), Chen et al. (18), Forsatkar et al. (22)*.

5 *Tahmasebi-Kohyani et al. (85), Kenari et al. (23), Palermo et al. (86), Fu et al. (25), Fuchs et al. (87)*.

6 *Küçükbay et al. (88), Betancor et al. (89), Long et al. (90), Izquierdo et al. (11), Kumar et al. (9)*.

### Amino Acids

It has been described that stressful husbandry conditions affect amino acid metabolism in fish (45, 91) and under some stress situations an increase in the requirement of certain essential amino acids occurs, which is probably related with the synthesis of proteins, and other compounds related with the stress response (92). The role of specific amino acids and their metabolites on key metabolic pathways that are necessary for growth, immunity or resistance to environmental stressors and pathogens have been already reviewed in fish (92–94). Thus, amino acids not only serve as constituents of proteins and energy sources, but also can be converted into important biochemically active substances *in vivo*.

Arginine is the precursor for the synthesis of nitric oxide (NO) and polyamines in higher vertebrates. In fish, NO production plays an important role in cellular defense mechanisms and has been demonstrated in stimulated macrophages in fish (56). Moreover, dietary arginine can increase some innate immune mechanisms and disease resistance of fish following challenge with Phdp (*Photobacterium damselae piscicida*) (56).

Branched-chain amino acids (BCAA: leucine, isoleucine and valine) have an important role in regulating protein synthesis in skeletal muscle, being leucine the most effective in the regulation of this process (95). An increased proteolysis activity is usually observed in fish under stressful situations, together with a decrease in plasma levels of BCAA (91, 96). Therefore, dietary supplementation with BCAA, especially leucine, appears to be a promising tool to mitigate negative effects of stress in fish.

Tryptophan (Trp) is an essential amino acid with important roles in the regulation of the stress response. It can be converted to serotonin (5-hydroxytryptamine, 5-HT) and melatonin (97). Nevertheless, over 95% of the ingested Trp is catabolized primarily in the liver via kynurenine pathway and produces niacin, pyruvate and acetyl-CoA as the final products (98). Brain 5-HT is involved in the control of the HPI axis in fish and a correlation between brain 5-HT activity and plasma cortisol levels has been observed (99). Indeed, tryptophan directly or indirectly participates in a wide array of physiological pathways, as recently reviewed by Hoseini et al. (94). In fact, fish under stressful husbandry conditions dropped free tryptophan levels in the plasma compared to control specimens (45, 91). Therefore, dietary tryptophan supplementation seems to be a promising nutritional strategy for health management in aquaculture.

Tyrosine is a common precursor for important hormones and neurotransmitters, including thyroxine, triiodothyronine, epinephrine, norepinephrine, dopamine, and melanin. These molecules have important important roles during stress response in fish, and thus tyrosine could profoundly influence pigmentation development, feed intake, growth performance, immunity, and survival of fish (93). It has reported that plasma free tyrosine concentrations increase during acute stress responses, suggesting tyrosine importance during stress response (96, 100).

Methionine also plays an important role in the antioxidant and immune status of animals as the precursor of cysteine, which in turn is required for the synthesis of glutathione and taurine (101). Some studies have reported changes in plasma levels of methionine in stressed fish compared to control specimens after both acute and chronic stressful conditions (45, 96, 100). Methionine metabolism can be directed to three pathways with health implications: (i) it provides s-adenosylmethionine that is then decarboxylated and turned into an aminopropane donor that fuels polyamine turnover (102), (ii) s-adenosylmethionine is directly involved in methylation of several cell constituents such as DNA, adrenergic, dopaminergic and serotonergic molecules (93); (iii) it leads to the transsulfuration pathway that ends up in the formation of glutathione from homocysteine (103). Therefore, an eventual increase in the requirement of methionine in fish under stressful conditions should be carefully considered.

Although the dietary protein is not a dietary additive, that is a key source for obtaining amino acids with relevant role in the stress response. In this sense, the effects of dietary protein (with no details on amino acid composition) concentration and its relation to lipid/carbohydrate content in fish have been widely studied, focusing on the nutritional issues (see Introduction). Regarding stress response, some of them have included stress markers, searching the optimum protein content to improve fish health and welfare (14, 16, 17, 47). The endocrine processes are not described in these works in detail (focused on nutrition), though it is supposed that the effects on stress response are based on amino acid content of those experimental diets.

### Vitamins

Vitamin C has been the object of the first works on stress attenuation through vitamin supplements, in both fish and superior animals (60, 104, 105). Moreover, from a nutritional perspective, the vitamin C content in fish feed is crucial since they are not able to synthesize it due to the lack of the enzyme L-gulonolactone oxidase, which is necessary to convert L-gulonic acid into vitamin C (106).

Its physiological role related to stress is based on the steroidogenesis inhibition through peroxidation of polyunsaturated lipids and the enhancement of the immune system (107–110). However, the effect of this supplement on the cortisol biosynthesis could not be demonstrated in fish (60, 111). Over 10 years later, Trenzado et al. (64) kept supporting this lack of connection between cortisol secretion and vitamin C. Nevertheless, Liu et al. (20) reported the beneficial immunomodulatory and antioxidant effects of vitamin C in stressed fish, stating that dietary ascorbic acid supplements alleviate chronic stress effects. In this sense, Imanpoor et al. (21) have recently demonstrated that vitamin C is a beneficial dietary supplement for improving the growth performance, survival, skeletal development and resistance to salinity stress of common carp fry. In spite of being object in many studies, there is not a general statement on the beneficial effects on vitamin C on the stress resistance, though no study indicates negative consequences of this feed supplement.

Vitamin E is required to maintain flesh quality, immunity, the normal resistance of red blood corpuscles to hemolysis, the maintenance of normal permeability of capillaries, and heart muscle (112, 113) Similarly to vitamin C, vitamin E effects on cultured fish welfare are based in its role as immunostimulant and antioxidant (61, 114, 115). This vitamin has been assayed successfully as inhibitor of cortisol secretion; in fact the most of works highlight this role, besides its stimulating effects on the immune system (13, 61, 63). Therefore, it seems that vitamin E could be a better stress alleviator than vitamin C, though the interaction of both vitamins with the stress system and cortisol and catecholamines secretion (endocrine and primary response) would not be clear yet.

Few works have studied the effects on other vitamins on the stress response, with no clear results regards stress alleviation. For instance, vitamin A is involved in metabolism, acting as a steroid hormone regulating growth through glycoprotein and glycosaminoglycan synthesis, as well as by modulating cell differentiation (67). In spite of those key physiological roles, Guimarães et al. (67) have reported that vitamin A does not provide any protection against cold-induced stress in fish. In this sense, Miao et al. (66) have demonstrated that, contrarily to the objective of the above works, long-term high doses of vitamin D_3_ lead to chronic stress and weaken the disease resistance. Therefore, the role and/or effects of vitamins different to C and E on the fish stress response are still unknown.

### Lipids and Fatty Acids

The study of the effects of dietary lipids on stress response, based on endocrine markers is relatively recent. Although some previous works dealt with the stress response in fish fed different lipid content, these used other markers as mortality, and oxygen consumption (116–118) One of the first trials including endocrine effects did not report promising results since no evidence on the relation between dietary lipid content and stress response was found (73). However, several successful works in this subject were published later (74, 76).

The importance of lipids in stress response is based on the formation of eicosanoids, particularly prostaglandins. Concretely, the Arachidonic Acid (ArA) can transform into eicosanoids, acting as endocrine, paracrine and/or autocrine modulators of secretory mechanisms in various organs (74). It has been stated that prostaglandins can modulate the sensitivity of the hypothalamus–pituitary–adrenal (HPA) axis in mammals and alter the release of cortisol and corticosterone in the stress response (119–121). In fish the interaction between HPI (hypothalamus–pituitary–interrenal) axis (equivalent to mammal HPA axis) response and dietary ArA has also demonstrated (122, 123). That is the reason which the most of studies on lipids and stress have focused in the dietary ArA as stress-attenuating biomolecule.

Mainly due to its key nutritional role, other fatty acids like docosahexanoic and eicosapentanoic acids (DHA and EPA) have been studied. Similarly, it has stated that several HUFAs (highly unsaturated fatty acids), for instance EPA, are also eicosanoid precursors. Besides eicosanoids, more fundamental processes like alterations in membrane properties and cellular signal transduction are supposed to contribute to the consistent effects of dietary DHA/EPA on growth, stress resistance and certain immune responses (80). Nevertheless, the knowledge of the interaction between HUFAs and HPI axis and cortisol secretion is very limited. Ganga et al. (124) have suggested that the oxygenated products of cyclooxygenase (COX) and lipoxygenase (LOX) derived from ArA, EPA, and DHA, respectively, may be major players in this regulation.

Besides HUFAs studies, the effects of dietary marine lecithine (mainly phospholipids) on stress response in fish have also reported (78). Phospholipids are known to facilitate digestion and absorption of lipids and other nutrients, form the structure of cellular membranes and support hyperplastic growth and may serve critical roles as the prevailing carriers of bioactive long-chain polyunsaturated fatty acids (LC-PUFA) and precursors to other physiologically active molecules (125). In fact, Trushenski et al. (78) stated that amending feed formulations with marine-origin phospholipid appears to be a practical approach to improve growth and stress tolerance in fish.

Astaxanthin (carotenoid) has also assayed as fish stress modulator and it has been reported that improves the acute overcrowding stress resistance though reduces the weight gain, CAT (catalase), and lysozyme activities (24). The anti-oxidative capacities of this compound are already known (126), though its relation to cortisol secretion decrease was not elucidated in that work.

### Prebiotics

The use of dietary carbohydrates to mitigate stress in fish has not been studied in deep. In fact, these biomolecules has been studied in a few works since some prebiotics are composed of them (22, 84, 127). Mannan-oligosaccharides (MOS) are one of the most studied prebiotics in fish, stating that improves growth, feed conversion, stress resistance, and immune function (128–130). The way which MOS act on the HPI axis has not been studied, though it is probable that the stress reduction is a consequence of the general fish welfare improvement. Therefore, probably the stress attenuation is not related directly to the consumption of these additives or their derived biomolecules.

### Nucleotides

Nucleotides refer to a group of biochemical substances (a purine or a pyrimidine base, a ribose or 2-deoxyribose sugar and one or more phosphate groups) with different physiological roles and biochemical functions since they are involved, for instance, in the vital cell function and metabolism, biosynthetic pathways, or mediating energy metabolism and cell signaling (131, 132). Dietary nucleotides are considered non-essential since neither prevailing biochemical malfunctions nor classical signs of deficiency are developed in endothermic animal models, and also due to the high rates of their *de novo* synthesis (e.g., RNA and DNA) that takes place in the human body, compared to the actual intake (133). The modulatory effects of dietary nucleotides on lymphocyte maturation, activation and proliferation, macrophage phagocytosis, immunoglobulin responses, gut microbiota as well as genetic expression of certain cytokines have been reported in endothermic animals (134).

The roles of nucleotides and metabolites in fish diets have been studied for almost 20 years, and most research has shown rather consistent and encouraging beneficial results in health management of both marine and freshwater fish. Li and Gatlin (132) reviewed the influence of dietary nucleotides on innate and adaptive immunity in fish and also suggested that dietary nucleotides would support lymphoid tissues that have limited “*de novo*” synthesizing capacity. Ringø et al. (135) recently pointed out that exogenous nucleotides have shown great potential as dietary supplements to enhance immunity and disease resistance of fish produced in aquaculture. Research on dietary nucleotides in fish has shown they may improve growth in early stages of development, alter intestinal structure, increase stress tolerance as well as modulate innate and adaptive immune responses (135). Despite occasional inconsistency in physiological responses, dietary supplementation of nucleotides has shown rather consistent beneficial influences on various fish species. In fact, fish fed nucleotide supplemented diets generally have shown enhanced resistance to viral, bacterial and parasitic infection (135, 136). However, little attention has been paid to the role of dietary nucleotides as stress-attenuating additives from an endocrine perspective.

### Minerals

The importance of mineral nutrition in relation to skeletal metabolism and health in fish have been described by Lall and Lewis-McCrea (137). Most available literature on mineral nutrition have aimed at determining optimum levels in diets for fish, and particular emphasis have been paid in early nutrition (11). Therefore, much effort needs to be taken to look at specific mineral requirements during adverse farming conditions to optimize aquaculture profitability. It seems clear that organic and inorganic selenium are the most frequent minerals assayed in order to reduce stress in fish (11, 88–90, 138). Selenium is cofactor in the antioxidant enzyme glutathione peroxidase (GPx), playing a crucial role in the oxidative stress (139). Therefore, the studies are focused on the oxidative stress response, instead of the endocrine one. All works have stated the beneficial effects of Se supplements on stress resistance due to its antioxidant action, and only Long et al. (90) have demonstrated, in addition, their effects on the inhibition of cortisol secretion. Manganese and zinc also have been tested (11). Similar to selenium, their roles as cofactors in several essential enzymes have been related to stress parameters attenuation, mainly those related to oxidative stress.

## Endocrine and Neuroendocrine Effects Along Species

The most of endocrine responses in the literature are based on plasma cortisol analysis, though the use of molecular markers and other hormones is progressively growing (see previous sections). The wide diversity of fish species (over 38), and additive type used make very difficult to analyse the effects of an only additive along the species. By that reason, a previous classification according to taxonomy or other features is appropriate to compare the effects of additives along species (Figure 1).

### Marine Species

The Table 2 shows an overview on the works on stress attenuation with dietary additives in marine species. The intensively cultured species have been used in the most of experiments, such as gilthead seabream (*Sparus aurata*), European seabass (*Dicentrarchus labrax*), Senegal sole (*Solea senegalensis*), and turbot (*Scophthalmus maximus*). Sometimes there are contradictory results for the same species and additive (74, 150), although the clear different responses are usually derived from distinct species, hence those responses are probably species-specific.

**Table 2:** General overview on the effects of dietary additives in marine fish submitted to stressful conditions.

**Additive**	**Fish species**	**Stress condition/treatment**	**Feeding time, days**	**Test doses**	**Main effects on physiology and productivity**
Arginine	*Solea senegalensis*^1^	Repeated daily handling (air exposure)	14	4.4–6.9 g 16 g^−1^ N	↑ ROS; ↑ NO
	*Scophthalmus maximus*^2^	Repeated handling (air exposure) every other day	15; 60	6–11 g 16 g^−1^ N	↓ Cortisol after 15 days ↑ ROS, plasma NO and ACH50 after 60 days ↑ Lysozyme after 15 and 60 days No effect on growth
Tryptophan	*Epinephelus coioides*^3^	Cohabitation for 10 days	10	0–1%	↓ Cannibalism rate ↑ Brain 5-HT contents ↓ Final weight
	*Gadus morhua* ^4, 5, 6^	Cohabitation for 7 days	7	2,8%	↓ Aggressive behavior
		Air exposure (3 min)Thermal shock (from 10 to 15°C in 30 min)	7	0.26–1.62%	↓ Cortisol and glucose in plasma of air exposed fish
		Confinement stress (i.e., lowering of water level) for 30 min	7	0.4–1.58%	↓ Cortisol in plasma in a dose dependent manner
	*Totoaba macdonaldi*^7^	Handling (chasing with a net for 45 min)Hypoxia (1 mg oxygen /L during 45 min)	21	0.5–2.3%	↑ Cortisol levels in fish submitted to handling and hypoxia ↓ Telencephalic 5-HT content in stressed specimens
	*Dicentrarchus labrax*^8^	Inflammatory insult (intraperitoneal injection with an inactivated pathogen)	14	1.12–2.24 g 16 g^−1^ N	↑ Cortisol levels at 24 h after injection
	*Argyrosomus regius*^9^	Air exposure (3 min)Confinement and netting (3 min)	7	0.07–0.11%	↓ Plasma protease activity in fish submitted to air exposure (after 6 h) or confinement and netting (after 1 h) ↑ Plasma bactericidal activity in air exposed fish after 1 h
	*Solea senegalensis*^10^	High density (31 kg/m^2^)	39	0.44–2.05%	↑ ACH50 in plasma ↑ Disease resistance
Methionine	*Sparus aurata*^11^	Hypoxia (2.8 mg oxygen /L during 5 h)	30	control; control + 0.3%	↓ Lactate in plasma ↓ SOD isoforms (Mn-SOD and CuZn-SOD) in liver
	*Dicentrarchus labrax*^8^	Inflammatory insult (intraperitoneal injection with an inactivated pathogen)	14	2.57–4.95 g 16 g^−1^ N	↑ Cortisol levels at 24 h after injection
Synergistic effects of amino acids	*Solea senegalensis*^12^	Repeated weekly handling (air exposure)	14; 28	Different amino acid mix	↓ Glucose and lactate after 14 days ↑ Lysozyme activity after 14 days ↑ Brain dopamine levels after 28 days
	*Solea senegalensis*^13^	High density (12 kg/m^2^)	18	Different amino acid mix	↓ Cortisol, glucose and lactate ↑ ACH50, lysozyme and peroxidase levels in plasma
Vitamin C	*Sparus aurata*^14^	High density (12 Kg/m^3^)	63	control; control + 0.025%	↓ Plasma lysozyme levels No effect on growth
	*Sebastes schlegelii*^15, 16^	Exposure to hexavalent chromium (i.e., 120 and 240 mg/L)	14; 28	0.01–0.0 4%	↓ Plasma cortisol levels only at 14 days ↓ Chromium accumulation in blood, kidney, liver, gut, gills and muscle ↑ Haematocrit
Vitamin E	*Sparus aurata*^14^	High density (12 Kg/m^3^)	63	control; control + 0.025%	↓ Plasma lysozyme levels ↑ ACH50 levels in plasma No effect on growth
	*Huso huso*^17^	Netting and air exposure (i.e., 1.5 min)	48	0.1–0.14%	↓ Plasma glucose levels ↑ WG
	*Takifugu obscurus*^18^	Exposure to ammonia-nitrogen for 48 h (i.e., 100 mg/L)	60	0.00023–0.03116%	↑ Expression levels of HSP, Mn-SOD, CAT and GR ↓ ROS in blood ↑ WG, SGR
ArA	*Sparus aurata*^19, 20, 21, 22, 23^	Daily salinity stress (fluctuating salinity over 24 h, from 25 to 40% and back to 25%)	20; 32	0.059–0.586% live prey DW	↑ Whole-body cortisol levels
		Air exposure for 90 s	28; 50	0.15–0.75% *Artemia* DW	↓ Whole-body cortisol levels ↑ Growth
		Confinement: 5·min of submersion in dip-net	18	0.9–2.4%	↓ Plasmacortisol levels
		Crowding stress (43–49 kg/m^3^)	240	0.2–1.11% FA	↓ Plasma cortisol levels
		Crowding stress (90–100 kg/m^3^)	72	0.13–0.31% TFA	↓ Plasma cortisol and glucose levels ↓ Gene expression in cell- and tissue-repairing markers, antioxidant enzymes, nuclear receptors and transcription factors
	*Solea senegalensis*^24, 25, 26^	Air exposure (2 min)	14	0.1–2.3% *Artemia* DW	↑ expression levels of PPARα and PEPCK
		Chasing stress test consisting of 5 min net chasing	84	0.5–0.8% TFA	↑ Expression levels of glucocorticoid receptor 1 and 2 in liver
				0.5–0.8% TFA	↑ Expression level of genes related to defensive response against virus, antigen differentiation and cytokines ↑ Final weight
	*Dicentrarchus labrax*^27^	Handling 20 larvae per tank out of the water in a scoop net for 1 min	14	0.3- 1.2%	↓ Gene expression of StAR and CYP11β ↑ Expression level of genes related to glucocorticoid receptor complex
EPA	*Solea senegalensis*^25, 26^	Chasing stress test consisting of 5 min net chasing	84	5.6–12%TFA	↑ Expression levels of glucocorticoid receptor 1 and 2 in liver
				5.6–12%TFA	↑ Expression level of genes related to defensive response against virus, antigen differentiation and cytokines ↑ Final weight
DHA	*Solea senegalensis*^25, 26^	Chasing stress test consisting of 5 min net chasing	84	4.9–11.1%TFA	↑ Expression levels of glucocorticoid receptor 1 and 2 in liver
				4.9–11.1%TFA	↑ Expression level of genes related to defensive response against virus, antigen differentiation and cytokines ↑ Final weight
MOS (prebiotic)	*Dicentrarchus labrax*^28^	Confinement stressor (25 kg/m^3^) Infection (intraperitoneal injection with (10^7^ cfu *Vibrio anguillarum*/ml)	60	0–0.4%	↓ Plasma cortisol levels in infected and stressed and infected groups ↑ Plasma cortisol levels in stressed groups ↓ Side-effects of stress on microflora profiles
	*Scophthalmus maximus*^29^	Handling procedure (combination of capture, netting/ transfer, and crowding)	112	0–0.6%	↓ Plasma cortisol and glucose levels at 1 h following acute stress
Nucleotide (Optimum)^*^	*Sciaenops ocellatus*^30^	Confinement stress (transfer of 3 fish from 110 L aquaria to 0.4 L for 15 min)	42	0–0.2%	No changes in plasma cortisol levels No effect on growth
Commercial nucleotides	*Gadus morhua*^31^	Acute stress:Salinity: increase from 35 to 50% during 30 minTemperature: increase from 12 to 15°C for 1hAir exposure for 45 s	38	0.5–1 g/L (live prey enrichment)	↓ Survival after air exposure No changes in cortisol levels ↑ HIF-2α in whole larvae ↑ Growth
Nucleotide (Vannagen)^*^	*Solea solea*^32^	Catching, netting and hand-sorting for 1 min	56	0–0.04%	↓ Plasma cortisol and glucose levels at 1 and 4 h following acute stress ↓ Brain cannabinoid receptor 1A and 1B mRNA expression at 4 h following acute stress
	*Scophthalmus maximus*^29^	Handling procedure (combination of capture, netting/ transfer, and crowding)	112	0–0.2%	↓ Plasma cortisol and glucose levels at 1 h following acute stress
Selenium (inorganic source–NaSe)	*Sparus aurata*^33^	Multiple stressful situations: persecution, handling and confinement for 2 h.	63	0.00002%	↓ Plasma cortisol levels at 2 h following acute stress
Selenium (organic source–SeMet)					

*5-HT, Serotonin; ACH50, Alternative Complement Pathway; ArA, Arachidonic Acid (20,4n-6); CAT, Catalase; CYP11β, 11β-hydroxylase; DHA, Docosahexaenoic Acid (22,6n-3); DPH, Days Post-Hatch; DW, Dry Weight; EPA, Eicosapentaenoic Acid (20:5n-3); GR, Glutathione Reductase; HIF, Hypoxia Inducible Factor; HSP, Heat-Shock Proteins; MOS, Mannan Oligosaccharides; NO, Nitric Oxide; PEPCK, Phosphoenolpyruvate Carboxykinase; PPARα, Peroxisome Proliferator-Activated Receptor Alpha; ROS, Reactive Oxygen Species; SGR, Specific Growth Rate; SOD, Superoxide Dismutase; StAR, Steroidogenic Acute Regulatory Protein; TFA, Total Fatty Acids; WG, Weight Gain*.

1 Costas et al. (56);

2 Costas et al. (140);

3 Hseu et al. (141);

4 Höglund et al. (142);

5 Herrera et al. (8);

6 Basic et al. (143);

7 Cabanillas-Gámez et al. (6);

8 Azeredo et al. (7);

9 Gonzalez-Silvera et al. (144);

10 Azeredo et al. (145);

11 Pérez-Jiménez et al. (146);

12 Costas et al. (147);

13 Costas et al. (57);

14 Montero et al. (114);

15 Kim et al. (148);

16 Kim and Kang (149);

17 Falahatkar et al. (65);

18 Cheng et al. (68);

19 Koven et al. (150);

20 Van Anholt et al. (74);

21 Van Anholt et al. (75);

22 Ganga et al. (151);

23 Pérez-Sánchez et al. (152);

24 Alves Martins et al. (77);

25 Benítez-Dorta et al. (153);

26 Montero et al. (154);

27 Montero et al. (155);

28 Torrecillas et al. (84);

29 Fuchs et al. (87);

30 Li et al. (156);

31 Lanes et al. (157);

32 Palermo et al. (86);

33 *Mechlaoui et al. (158)*.

* *Optimum®, Vannagen® supplied by Chemoforma (Augst, Switzerland)*.

#### Amino Acids

Fish present additional amino acid requirements when submitted to stressful rearing conditions, due to either increased energy demands or for the synthesis of stress-related proteins and other compounds related with the stress response (92). In this context, increasing evidence suggests the possibility of mitigating the negative physiological effects attributed to stress (see previous sections) by altering dietary amino acid levels.

Studies with flatfish species gathered some knowledge regarding the role of dietary arginine during chronic stressful conditions. It was observed that duration (e.g., 14/15 or 60 days) of handling procedures induced different responses in some innate immune parameters of Senegal sole and turbot (56, 140). While repeated acute stress reduced NO levels in turbot at both sampling times, a positive synergistic effect between dietary arginine and stress was observed in sole. Handling stress also decreased cellular ROS in both flatfish species, a fact that seems to be counteracted by dietary arginine after 60 days of feeding in turbot. Depending on the duration and severity of the stressor, increased glucocorticoid levels may enhance innate and adaptive immune responses while similar hormone levels may suppress immune function. Therefore, the suppressive effect of stress on the innate immune system is highly disputable and does not necessarily translate in decrease resistance to infection, as already suggested elsewhere (2, 159).

Tryptophan has been the central character in many stress mitigation studies in marine fish. A recent review has covered the involvement of tryptophan in 5HT and melatonin-mediated functions, along with its participation in the regulation of the immune system and its role as an antioxidant and antitoxic agent in fish (94). In general, a positive effect is usually attributed to tryptophan nutrition in stressed animals. In marine fish, a number of studies have already tested the effects of dietary tryptophan under both acute and chronic stressful conditions. In those works, feeding strategies varied from 7 to 39 days, being shorter times more frequently used prior to an acute stress event. Indeed, 7 and 10 days of tryptophan treatment decreased aggressive behavior and cannibalism rate in juvenile Atlantic cod (*Gadus morhua*) and grouper (*Epinephelus coioides*), respectively (141, 142). However, fish fed tryptophan supplemented diets and reared under non-stressful conditions seem to cope differently with the stress imposed depending on feeding time. For instance, Atlantic cod fed tryptophan supplemented diets for 7 days decreased plasma cortisol and glucose levels immediately after air exposure, whereas totoaba (*Totoaba macdonaldi*) and European seabass fed tryptophan surplus increased plasma cortisol levels after handling (chasing with a net for 45 min) and hypoxia (1 mg oxygen /L during 45 min) or an inflammatory insult, respectively, (6–8). In contrast, Senegalese sole juveniles fed tryptophan supplemented diets showed a trend to decrease plasma cortisol levels when reared at high stocking densities (i.e., 31 kg/m^2^), which translated in enhanced disease resistance after 39 days of feeding.

Methionine also seems to play a role in the stress response probably due to its important role in the transsulfuration pathway. In a study with gilthead seabream, fish fed dietary methionine surplus for 30 days decreased plasma lactate levels and the superoxide dismutase (SOD) isoenzymatic profile (Mn-SOD and CuZn-SOD) in liver after hypoxia treatment (i.e., 2.8 mg oxygen /L during 5 h) (146). However, European seabass fed a methionine supplemented diet for 14 days showed the opposite trend with increased plasma cortisol levels at 24 h after an inflammatory insult (7).

While most research focused on the effects of individual dietary amino acids supplementation in fish submitted to stressful conditions, some other works increased the amount of digestible protein and therefore the availability of certain amino acids (AA). For instance, Costas et al. (147) observed that a slight increase in the availability of some dietary amino acids (arginine, phenylalanine, and tryptophan) may have a significant impact on amino acid metabolism, as indicated by changes in plasma amino acid levels compared to chronically stressed treatments. Therefore, providing those key AA in the diet may represent a metabolic advantage during predictable stressful events (e.g., handling and overcrowding associated to grading procedures), which may have a significant effect on growth and welfare in the longer term. Those effects on metabolism appear to be stronger after 14 days compared to 28 days of feeding, as indicated by the reduction of plasma glucose and lactate levels. Still, 28 days of feeding appear to have some effect on other processes related to the stress response. In a similar study, Senegalese sole exposed to a high density for 18 days and fed a diet with an increase in some key AA, counteracted the negative effects of chronic stress and increased plasma complement, lysozyme and peroxidase activities compared to their counterparts fed the control diet (57).

#### Vitamins

Vitamins have been demonstrated to improve immune responses to infection by affecting the proliferation and migration of immune cells such as phagocytic cells, equipping the fish with an improved resistance to diseases (160). Although vitamin levels required for fish are influenced by several factors such as environmental factors, few studies have gathered deep knowledge on the modulatory role of vitamins during stressful rearing conditions. Low levels of vitamin E in the diet depleted alternative complement pathway activity and non-specific haemaglutination whereas plasma cortisol basal levels were enhanced without a stressor influence (61). Moreover, this study concluded that fish fed a vitamin E-deficient diet presented lower stress resistance.

Positive effects of dietary vitamin E supplementation have observed in several marine fish species submitted to stressful conditions. For instance, pufferfish (*Takifugu obscurus*) fed vitamin E supplemented diets increased relative expression levels of HSP, Mn-SOD, CAT, and GR whereas ROS levels in blood decreased after acute exposure to ammonia nitrogen (100 mg/L) for 48 h (68). Moreover, beluga (*Huso huso*) submitted to netting and exposed to air for 1.5 min decreased post-stress plasma glucose levels when fed diets supplemented with vitamin E (65). In general, the stress response of the belugas observed in this study was relatively low, and the authors hypothesized that it could be related to greater resistance and/or weaker physiological responses to handling stress in that species. Montero et al. (114) observed that gilthead seabream reared at an initial stocking density of 12 Kg/m^3^ (final density: 40 Kg/m^3^) increased plasma cortisol and serum lysozyme levels whereas serum ACH50 values decreased. Those fish fed on Vitamin C or a Vitamin E supplemented diets did not change cortisol levels but a decrease in lysozyme was observed, in contrast to the augmentation in serum ACH50 from fish fed the vitamin E supplemented diet.

#### Lipids and Fatty Acids

It has been reported that dietary lipids can affect the fish stress response, measured as the ability to cope with different stressful situations (74, 75, 151, 152). However, the specific effect of individual fatty acids on the physiological response to stress is still poorly understood, particularly in terms of the modulatory role of fatty acids in the activation of the HPI axis. Arachidonic acid has played a central role in recent studies concerning research on the modulatory roles of dietary fatty acids in the fish stress response. The regulatory role of ArA on the ACTH-induced release of cortisol has been described *in vitro* for gilthead seabream by Ganga et al. (122) and for European seabass by Montero et al. (123). Seabream juveniles fed diets with a high inclusion of vegetable oils (e.g., linseed, rapeseed and palm oils), which translated in a drop in dietary ArA content, increased plasma cortisol levels following an acute overcrowding stress (124, 152). Similarly, feeding an ArA-supplemented diet to gilthead seabream juveniles for 18 days was effective to substantially diminish the cortisol response after net confinement, compared to fish fed a diet containing a low ArA level (74). Benítez-Dorta et al. (153) observed an increase in the level of mRNA expression in glucocorticoid receptor genes after a chasing stress in Senegalese sole juveniles fed a fish oil-based diet (i.e., with high ArA levels) compared to counterpart fed a vegetable oil-based diet (i.e., with low ArA levels). This decreased response to stress was in line to what was found in gilthead seabream larvae submitted to air exposure which showed a considerable drop in peak cortisol levels 28 or 50 days after hatching when they were fed ArA-enriched *Artemia* nauplii (75). In this sense, European seabass fed dietary ArA supplementation decreased the level of expression of P450 11β-hydroxylase (enzyme related cortisol-synthesis), which translated in an increased survival after an activity test consisting of handling procedures and transfer to a new tank (155). In contrast, pre-metarmophosing gilthead seabream larvae daily exposed to fluctuations in salinity increased whole-body cortisol levels when fed ArA-enriched *Artemia* metanauplii for 12 days, which translated in a decreased in survival at 32 days after hatching (150). These findings contrast with the survival-promoting effect of high dietary ArA in larvae exposed only to handling and having relatively low basal cortisol levels. These authors hypothesized that a clue for those physiological mechanisms could be found in mammalian studies where not only prostaglandin E2 synthesized from the cyclooxygenase enzymes but other ArA metabolites, such as leukotrienes produced from the lipoxygenase enzyme system, also play an important role in ACTH secretion and adrenal steroidogenesis (121, 161).

The fish stress response is therefore nutritionally regulated, and in fact a study with gilthead seabream highlights that the magnitude and persistence of high plasma cortisol levels after overcrowding exposure are dependent on the source of dietary oils (124). Indeed, dietary oils source and, hence, dietary essential fatty acids clearly affected resting levels of glucocorticoid receptor genes expression in Senegalese sole juveniles and larvae and European seabass larvae (77, 153, 155). Moreover, Benítez-Dorta et al. (153) observed and increase in the level of mRNA expression in glucocorticoid receptor genes after a chasing stress in Senegalese sole juveniles fed a fish oil-based diet (i.e., with high ArA levels) compared to specimens fed a vegetable oil-based diet (i.e., with low ArA levels). Those experimental conditions also seemed to affect the Senegalese sole immune response to chasing stress (154).

ArA effects on the stress resistance seem to depend on ArA doses, species or type of stress, but these effects are also dependent on the abundance of n-3 LC-PUFA such as EPA and DHA, since these fatty acids are also essential for stress resistance (162, 163). For instance, ArA and particularly EPA promoted cortisol production in gilthead seabream interrenal cells (122). Moreover, Alves Martins et al. (164) hypothesized that the abundance of ArA relative to EPA (or their oxidized derivatives) in Senegalese sole fed a high ArA/EPA diet could influence StAR (Steroidogenic Acute Regulatory) protein, increase cortisol production and ultimately imply higher energy expenditure to cope with stress.

#### Prebiotics

The effects of prebiotics supplementation in relation to stress response have scarcely been studied in marine fish. For instance, Torrecillas et al. (84) observed that European seabass fed Bio-Mos® (Alltech, Inc., Nicholasville, KY, USA) dietary supplementation at 0.4% for 60 days reduced plasma cortisol levels in response to a challenge with *Vibrio anguillarum* (i.e., 107 cfu/ml) or to a combination of infection and confinement stress (25 kg/m^3^). In contrast, European seabass submitted to confinement stress alone and fed Bio-Mos® increased plasma cortisol levels following acute stress whereas a lower effect of stress on gut microbiota was found in those fish fed 0.4% Bio-Mos® during 60 days compared to stressed fish fed a control diet. Indeed, it has been already reported that mannan oligosaccharides (MOS) supplementation reinforces epithelial barrier, stimulates the immune system, promotes growth and feed efficiency and effectively enhances disease resistance in fish (130). In another study, Fuchs et al. (87) studied the effects of a 6% yeast (*Saccharomyces cerevisiae*) product consisting of 20% beta-1,3/1,6 glucan and 17% MOS (ProEnMune, ProEn Protein, and Energie GmbH, Soltau, Germany) in turbot juveniles. In contrast to that observed by Torrecillas et al. (84), it was observed a decrease in plasma cortisol and glucose levels at 1 h after acute stress. However, this decrease in both primary and secondary stress responses observed in stressed turbot could be attributed to a synergistic effect of both beta-1,3/1,6 glucan and MOS from yeast, thus making difficult a direct comparison on the effects of dietary MOS within marine fish species submitted to stressful conditions.

#### Nucleotides

Studies on different fish species reported that dietary nucleotide supplementation enhanced their resistance to parasites, bacteria and virus (136), while the effects of those particular additives on the marine fish stress response still remain to be studied in detail. For instance, a study on Atlantic cod larvae suggested that a nucleotide-enriched *Artemia* can benefit growth whereas those larvae appeared to be more susceptible to acute stress as evidenced by the lower survival rates and higher *hif-2*α transcript levels in whole larvae, although cortisol levels were not affected (157). Likewise, red drum (*Sciaenops ocellatus*) juveniles fed a nucleotide product (i.e., Optimun, Chemoforma, Basel, Switzerland), which contained cytidine-50-monophosphate, disodiumuridine-50-monophosphate, adenosine-50-monophosphate, disodium inosine-50-monophosphate, disodium guanidine-50-monophosphate, and RNA, did not change plasma cortisol levels in after a 15 min confinement stress test, a fact that could be linked to a high individual variation among fish (156). In contrast, turbot juveniles submitted to an acute stress (i.e., handling procedure consisting of a combination of capture, netting/transfer, and overcrowding from 13.3 to 32.4 kg m^−2^) and fed a product of purified yeast nucleotides for 112 days decreased both plasma cortisol and glucose levels at 1 h after acute stress. According to Palermo et al. (86), Senegalese sole fed a commercial source of nucleotides derived from yeast (Vannagen™, Chemoforma) for 8 weeks coped well with an acute stress challenge (i.e., catching, netting and hand-sorting for 1 min) and presented lower plasma cortisol and glucose levels than control fish. Those authors also reported a decrease in the mRNA expression level of brain cannabinoid receptors 1A and 1B in fish fed the nucleotides supplemented diet after acute stress, and suggested a putative nucleotides effect on the functional interaction between endocannabinoid signaling system and stress axis in fish, a fact that deserves further attention.

#### Minerals

Indeed, information regarding mineral nutrition in marine fish is still scarce, a lack of knowledge that seems to increase when assessing the stress response in fish. Selenium in particular is an essential trace element for fish (139), and therefore it plays an important role for growth and conservation of biological compounds, exerting protection against free radicals resulting from normal metabolism (165). An increase in dietary selenium supplementation (i.e., organic and inorganic forms) appeared to increase stress tolerance in gilthead seabream juveniles, as shown by the decreased plasma cortisol levels during the stress challenge in specimens submitted to acute stress (158). The later study reinforced the importance of dietary selenium supplementation on health and welfare in gilthead seabream, similarly to that reported for salmonid species (see section Minerals below).

### Salmonids

Atlantic salmon (*Salmo salmo*) and rainbow trout (*Onchorhynchus mykiss*) are the most studied salmonid species in the literature (Table 3). Contrarily to marine species, here it seems that stress responses are more consistent since, for the same species and additive, the results on stress parameters are not different among every work (55, 97, 167, 168).

**Table 3:** General overview on the effects of dietary additives in salmonids submitted to stressful conditions.

**Additive**	**Fish species**	**Stress condition/treatment**	**Feeding time, days**	**Test doses**	**Main effects on physiology and productivity**
Tryptophan	*Salmo trutta*^1^	Transfer to a new environment	7	0.22–0.06 Trp/LNNA	↓ Stress-induced anorexia
	*Oncorhynchus mykiss*^2^^,^^3^^,^^4^^,^^5^^,^^6^	Resident/intruder test	3; 7	0.15–1.5%	↓ Aggressive behavior in fish fed for 7 days
		Lowering the water level for 2 h	7	0.44–3.57%	↓ Adrenocorticotropic hormone and cortisol levels in plasma ↑ Brain serotonergic activity
		Lowering the water level for 2 h	3; 7; 28	0.044–0.357%	↓ Adrenocorticotropic hormone and cortisol levels in plasma after 7 days of feeding
		Daily social interaction for 1 h followed by a resident/intruder test after 1 week	7	0.044–0.357%	↓ Aggressive behavior ↓ Cortisol levels in plasma
		Lowering the water level for 2 h	7	0.044–0.357%	↓ Cortisol and melatonin levels in plasma
	*Salmo salar*^7^^,^^8^	Confinement for 30 min at days 1, 2, and 10 after tryptophan treatment	7	0.4–1.58%	↓ Plasma cortisol levels at day 10 after tryptophan treatment
		Acute crowding stress for 1 h at days 8 and 21 after tryptophan treatment	7	0.44–1.2%	↓ Plasma cortisol levels at days 8 and 21 after tryptophan treatment
Vitamin C	*Salmo salar*^9^	Confinement for 2 h	161	0.0082–0.317%	↓ Plasma antibody titers at 43 days post-immunization
Vitamin E	*Oncorhynchus mykiss*^10^^,^^11^	High density (100 kg/m^3^)	42	0.00256–0.02756%	↑ MCV
		High density (80 kg/m^3^)	60	0.010475–0.060075%	↓ Cortisol and lactate levels in plasma ↑ SOD in liver ↓ MDA in liver ↑ SGR, WG, FI
Nucleotide (Optimum)^*^	*Oncorhynchus mykiss*^12^^,^^13^	Netting, air exposure for 30 s, and crowding at 100 kg/m^3^ for 3 h	56	0–0.2%	↓ Plasma cortisol levels in infected and stressed and infected groups ↑ Plasma cortisol levels in stressed groups ↓ Side-effects of stress on microflora profiles ↑ WG, FE
		High density (30 kg/m^3^)	45	0.2%	↓ Serum urea and ACH50 levels No effect on growth
	*Salmo trutta caspius*^14^	Netting, air exposure for 30 s, and crowding at 100 kg/m^3^ for 3 h	56	0.15–0.5%	↓ Plasma cortisol and glucose levels at 8 h following acute stress ↑ Final weight
		Transfer to salt water (18 g/L)		0.15–0.5%	↓ Plasma cortisol levels at 120 h following acute stress ↑ Final weight
Nucleotide (Maxi-Gen Plus)^#^	*Salmo salar*^15^	Smoltification process	122	0.05–0.60%	↓ Plasma cortisol levels ↑ WG, FI
Selenium	*Oncorhynchus mykiss*^11^^,^^16^^,^^17^	High density (80 kg/m^3^)	60	0.000035–0.000135%	↓ Serum lactate, ALP and ALT levels ↑ Hepatic GPx activity ↓ SOD activity in liver No effects on growth
		Acute stress for 7 days consisting of a combination of daily crowding and handling (i.e., netting and air exposure for 30 s) twice a day	70	0.00073–0.00074%	↑ ROS in blood ↑ Hepatic MDA ↑ Whole body copper
		High density (100 kg/m^3^)	84	0.00008–0.00011%	↓ MDA levels in serum and muscle ↓ Serum GPx activity ↓ HSP70 expression in muscle ↑ Final weight, FI

*ACH50, Alternative Complement Pathway; ALT, Alanine Aminotransferase; ALP, Alkaline Phosphatase; FE, Feed Efficiency; FI, Feed Intake; GPx, Glutathione Peroxidase; HSP70, Heat Shock Protein 70; LNNA, Large Neutral Amino Acids; MCV, Mean Corpuscular Volume; MDA, Malondialdehyde; ROS, Reactive Oxygen Species; SGR, Specific Growth Rate; SOD, Superoxide Dismutase; Trp, Tryptophan; WG, Weight Gain*.

1 Höglund et al. (44);

2 Winberg et al. (166);

3 Lepage et al. (167);

4 Lepage et al. (97);

5 Lepage et al. (168);

6 Lepage et al. (55);

7 Basic et al. (169);

8 Höglund et al. (170);

9 Thompson et al. (60);

10 Trenzado et al. (171);

11 Naderi et al. (172);

12 Tahmasebi-Kohyani et al. (85);

13 Yousefi et al. (173);

14 Kenari et al. (23);

15 Fu et al. (25);

16 Rider et al. (165);

17 *Küçükbay et al. (88)*.

* *Optimum® supplied by Chemoforma (Augst, Switzerland)*.

# *Maxi-Gen Plus® supplied by Canadian Bio-Systems Inc. (Calgary, AB, Canada)*.

#### Amino Acids

Research with salmonid species mainly studied the modulatory role of dietary tryptophan on the fish stress response, including aggressive behavior, to an acute stressful condition. Moreover, those studies particularly emphasized on the short-term effect of tryptophan treatment (e.g., 7 days). For instance, some recent findings showed that tryptophan administration can increase serotonergic activity by means of increased 5HT and/or 5HIAA (97, 167, 169); while others suggested a suppression in aggressive behavior and stress-induced anorexia (44, 166). In rainbow trout, a 7-day tryptophan treatment suppressed post-acute stress cortisol increase, a fact that appears to be modulated by serotonergic activity and ACTH release (97, 167).

In contrast, other researchers investigated if dietary tryptophan treatment may result in long-lasting effects on stress responsiveness. For instance, Atlantic salmon decreased post-acute stress cortisol levels at days 8, 10, and 21 following a 7-day period tryptophan administration (169, 170). The importance of tryptophan administration time on serotonergic activity and cortisol response has also been suggested for the rainbow trout (97). Still, there are no evidences for the effects of long-term dietary tryptophan administration on the stress response in salmonids, a fact that deserves further attention.

#### Vitamins

Few studies with salmonid species have focused on the modulatory role of vitamins during stressful rearing conditions. Thompson et al. (60) did not observe any evidence that dietary vitamin C (3.17 g/kg diet) can ameliorate the down regulation of the immune system that occurs following confinement stress in the Atlantic salmon, suggesting that vitamin C does not play a fundamental role in regulating the primary stress response in salmonids. In contrast, dietary supplementation of vitamin E (275.6 mg/kg diet) appears to enhance the MCV (Mean Corpuscular Volume) of rainbow trout reared at high density (i.e., 100 kg/m^3^) for 42 days (171). 138 also reported a positive effect of vitamin E supplementation (500 mg/kg diet) in chronically stressed rainbow trout for 60 days. In this study, dietary vitamin E reverted the negative effects of high density (i.e., 80 kg/m^3^) by decreasing plasma cortisol and lactate levels. Moreover, those fish also presented and enhanced SOD activity as well as a decrease in MDA (Malondialdehyde) in liver. A synergistic effect of dietary vitamin E supplementation with HUFA was also observed in chronically stressed rainbow trout with an increase of plasma cortisol after 42 days reared at high density (64). Those fish also showed an enhanced catalase activity in liver compared to their low density counterparts, a fact that could be related to the lipid-soluble character of vitamin E.

#### Nucleotides

Most studies concerning nucleotides nutrition in salmonids as a strategy to mitigate the negative effects of stress were performed with the same commercial additive (Optimun, Chemoforma, Augst, Switzerland). Rainbow trout fed diets containing 0.15–0.2% nucleotides from Optimun improved growth performance and several hematological and biochemical parameters, which translated in a significant reduction of plasma cortisol and glucose after exposure to acute handling and overcrowding stress (85). Leonardi et al. (174) also observed positive health-related effects in rainbow trout fed the same dietary additive at 0.03%, since those fish decreased plasma cortisol levels following challenge with infectious pancreatic necrosis virus. Furthermore, Caspian brown trout (*Salmo trutta caspius*) fed an Optimun supplemented diet (i.e., 0.25%) for 56 days decreased plasma cortisol and glucose levels after acute confinement and salinity stress (23). In contrast, rainbow trout fed an Optimun supplemented diet (i.e., 0.2%) for 45 days did not improve growth performance nor stressful condition in high density groups, which decreased serum ACH50 levels (173). Fu et al. (25) assayed diets supplemented with graded levels of Maxi-Gen™ Plus (Canadian Bio-Systems Inc., Calgary, AB, Canada) with Atlantic salmon during smoltification, showing that the hypo-osmoregulatory ability was gradually enhanced when the dietary inclusion level of Maxi-Gen™ Plus augmented from 0.05 to 0.20%, and from 0.20 to 0.60%. Moreover, an inclusion of 0.60% Maxi-Gen™ Plus in the diet resulted in lower plasma cortisol levels of smolting Atlantic salmon compared to fish fed the control diet, suggesting reduced stress levels in fish during smoltification and desmoltification.

#### Minerals

Depending on its chemical form, selenium is a trace element with a narrow range between requirement and toxicity for most vertebrates, and thus some studies were undertaken to assess and recommend safe limits regarding selenium nutrition in salmonids (175, 176). However, few studies have been conducted with salmonid species submitted to stressful conditions. Rainbow trout submitted to acute stressful situations for 7 days or to crowding conditions (100 kg/m^3^) for 86 days seem to increase selenium requirement for an optimal oxidative status (88, 165). In fact, Naderi et al. (172) reported a drop in serum lactate, alanine aminotransferase and alkaline phosphatase levels together with enhanced glutathione peroxidase activity in liver in rainbow trouts fed Se supplements under high density. Interestingly, in that study a positive synergistic effect between dietary organic selenium and vitamin E was observed, which translated in decreased serum cortisol levels as well as improved superoxide dismutase activity and low MDA levels in liver.

### Cyprinids

In this order more than 10 additives and seven species have been assayed (Table 4). The most of works have been focused on amino acids and vitamins. Only two works have dealt with minerals and carbohydrates (22, 90).

**Table 4:** General overview on the effects of dietary additives in cyprinids submitted to stressful conditions.

**Additive**	**Fish species**	**Stress condition/treatment**	**Feeding time, days**	**Test doses**	**Main effects on physiology and productivity**
Alanine and glutamine	*Cyprinus carpio*^1^	High density (80 g/L)	56	0–1%	↑ Serum IGF-I and insulin ↓ Serum glucagon ↑ GR gene expression ↑ WG
Tryptophan	*Labeo rohita*^2^	Thermal stress (34 and 38°C)	45	0–1.42%	↓ Blood glucose and serum cortisol ↓ AST and ALT activities ↓ LDH and MDH activities ↓ AchE, CAT, and SOD activities ↑ RGR, PER
	*Cirrhinus mrigala*^3^	Crowding stress (30 fish/75 L, 3-fold control group)	60	0–2.72%	↓ Blood glucose and plasma cortisol ↓ AST and ALT activities ↓ MDH activity ↑ AchE activity ↑ SGR, PER
Taurine	*Mylopharyngodon piceus*^4^	Crowding stress (100 g/L) for 24 h after experimental feeding	56	0–0.4%	↓ Serum glucose and cortisol ↑ Serum complement C3,lysozyme, SOD and glutathione ↑ WG
Vitamin C	*Cyprinus carpio*^5^	Salinity stress (0, 6 and 2 ppt)	48	0–0.1%	↓ Blood cortisol ↓ Skeletal malformations
Vitamins C + E	(*Notemigonus crysoleucas*)^6^	Vitamins C + E combinations and thermal stress (37°C)	119	0–0.000038% vit E0–0.000222% vit C	Different interactive effects ↑ACH50 No effect on growth
Vitamin E	(*Megalobrama amblycephala*)^7^	Crowding stress for 48 h (100 g/L)	60	0.1–0.6%	↓ Serum glucose and cortisol ↓ Serum ALT and lysozyme activities ↑ Serum proteins ↓ Hepatic MDA content ↑ HSP70 expression ↑ SGR
Vitamins mix (C, B1, B6, and E)	*Cyprinus carpio*^8^	Handling (confinement) stress: 2 cm water depth for 2 h	14	Different mixes	↓ Mucus immunoglobulins No effect on growth
Lipids	(*Notemigonus crysoleucas*)^9^	Crowding stress (4 cm water depth for 2 h)	42	4–13% different oils	No changes in cortisol response
MOS (prebiotic)	(*Danio rerio)*^10^	Starvation, live transport and tank cleaning	56	0–0.4%	↓ Cortisol and CRH gene expression
Selenium	(*Megalobrama amblycephala*)^11^	Nitrite exposure (15 mg/L for 96 h)	60	0–0.00005%	↓ Serum cortisol ↓ Hepatic MDA content ↑ SOD, CAT and GPx activities and transcriptions

*ACH50, Alternative Complement Activity; AchE, Acetylcholine Esterase; ALT, Alanine Aminotransferase; AST, Aspartate Aminotransferase; CAT, Catalase;; CRH, Corticotropin Releasing Hormone; GR, Glucocorticoid Receptor; HSP70, Heat Shock Protein 70 KDa; IGF-I, Insuline Growth Factor I; LDH, Lactate Dehydrogenase; MDA, Malondialdehyde; MDH, Malate Dehydrogenase; MOS, mannan-oligosaccharide; PER, Protein Efficiency Ratio; RGR, Relative Growth Rate; SGR, Specific Growth Rate; SOD, Superoxide Dismutase; WG, Weight Gain*.

1 Chen et al. (51);

2 Kumar et al. (49);

3 Tejpal et al. (46);

4 Tian et al. (52);

5 Imanpoor et al. (21);

6 Chen et al. (62);

7 Liu et al. (13);

8 Sándor et al. (69);

9 Lochmann et al. (73);

10 Forsatkar et al. (22);

11 *Long et al. (90)*.

#### Amino Acids

It seems clear that amino acid effects, concretely tryptophan (Trp) supplements, are consistent along cyprinid species. In this sense Kumar et al. (49) and Tejpal et al. (46) have reported significant cortisol secretion decreases after stress in rohu (*Labeo rohita*) and mrigal (*Cirrhinus mrigala*), respectively. In addition, abovementioned two studies papers have stated a growth enhancement after feeding Trp-enriched diets for 45–60 days. The amount of Trp in diet have been very similar in both papers, hence 1–1.5% Trp on dry matter basis is effective to attenuate the stress response in cyprinids. In addition, the stressors were different in both works, hence it seems that the stress response in cyprinids fed Trp supplements is enough consistent along species. Tejpal et al. (46) have also established a linear relation between Trp content and plasma cortisol for both stressed (overcrowding) and non-stressed rohus, and have used that mathematical equation to define the optimum Trp content (1.36%) for the highest stress attenuation.

Other amino acids like alanine (Ala) and glutamine (Gln) did not affect cortisol response in carp (*Cyprinus carpio*) though growth performance was significantly improved (51). Spite of the lack of cortisol response in this work, other hormones variations reflected the addition of dietary amino acids. In fact, IGF-I (Insulin-like Growth Factor I) and insulin significantly increased with dietary Ala-Gln supplementation under overcrowding stress. Therefore, the authors concluded that Ala-Gln supplements enhance the ability of fish resistance to overcrowding stress, which may contribute to the better regulation ability for hormone secretion on fish.

Regards dietary total protein, Habte-Tsion et al. (14) have studied the effects of different protein ratios (28–36%) in feed on the stress response in the blunt snout bream (*Megalobrama amblycephala*). Under thermal stress, the cortisol secretion was minimum in fish fed diet containing 32% dietary protein. This treatment also showed positive results in other immune and stress oxidative parameters. Additionally, the authors reported that the specific molecular mechanisms by which the optimum dietary protein level reduced the level of cortisol in high temperature stressed blunt snout breams need to be researched.

The relation between dietary lipid and protein contents, and stress response have also tested in cyprinids. In those cases, the role of dietary proteins seems to more decisive than lipids since golden shiners did not show significant differences in the endocrine stress response depending on dietary lipid level, meanwhile Habte-Tsion et al. (14) stated that the optimum protein content for decreasing the cortisol response significantly in blunt snout bream was 32%.

#### Vitamins

Vitamins C and E have been assayed in some Cyprinid species. The beneficial antioxidant properties and the reduction of cortisol response after stress are common results in recent studies (13, 21, 62, 66, 69). Moreover, these have reported other positive effects like immune system and growth enhancement, higher survival and lower skeleton abnormalities. However, several differences have been detected among species. It seems that the vitamin C requirements to improve stress resistance in carp is around 50 mg/Kg diet, while golden shiners (*Notemigonus crysoleucas*) need more than 98 mg/Kg (21, 62). Similarly, 600 mg/Kg diet of vitamin E are reported to be enough to reduce the post-stress cortisol secretion in blunt snout bream (*Megalobrama amblycephala*) (13), and Chen et al. (62) point that 38 mg/Kg diet is a suitable vitamin E concentration to improve stress resistance in the golden shiner. In those cases, the vitamin requirements for improving the stress response are clearly different along species, which could be expectable since those requirements are in that way from a nutritional perspective.

#### Prebiotics

The inclusion of prebiotics, particularly MOS (mannan-oligosaccharides), in the diet have also demonstrated to have stress-attenuating effects at endocrine level in cyprinids (22). Both cortisol secretion and CRH expression level were significantly reduced after feed deprivation stress in zebrafish fed MOS. In addition, the inclusion of MOS in the diet of zebrafish reduced some anxiety-like behaviors in fish submitted to feed deprivation. Those authors stated that all the physiological alterations were the results of alteration in intestinal microbiota, and the modulation of gut microbiota by MOS play a role in the stress reactivity of zebrafish.

### Other Freshwater Species

As in the other groups, amino acids and proteins are the most frequent substances assayed in these 11 different freshwater (excluding cyprinids) fish species (Table 5). This is the most heterogeneous group regards both species and stress response. Opposite endocrine stress responses have been described for every additive type in these species.

**Table 5:** General overview on the effects of dietary additives in other freshwater species submitted to stressful conditions.

**Additive**	**Fish species**	**Stress condition/treatment**	**Feeding time, days**	**Test doses**	**Main effects on physiology and productivity**
Tryptophan	*Brycon amazonicus*^1^	Aggressiveness test (resident-intruder)	7	0.94–3.76%	↓ Aggressiveness No effect on physiological stress markers
	*Cichlasoma dimerus*^2^	Normal experimental conditions	28	control; control + 2.1%	↓ Plasma cortisol ↓ Brain serotonergic activity No effect on growth
	*Acipenser persicus*^3^	Confinement (0.5 h)	5; 10; 15	0.28–0.78%	↓ Serum thyroid hormones ↑ Serum cortisol
	*Oreochromis niloticus*^4^	Crowding (50% water volume)+chasing (20 min)	7	0.48–4.45%	↑ Brain serotonin metabolites No effect on the HPI axis
Protein levels	*Oreochromis niloticus*^5^^,^^6^	Experimental conditions (different protein levels)	70	25–45%	↑ Serum glucose, proteins and lipids ↑ ALT and AST activities ↑ SGR, PER, FI
		Simulated haul	84	28–36%	No effect on physiological stress markers No differences for WG, FI ↓ FCR
	*Acipenser baerii*^7^	Experimental conditions (different protein levels)	70	38–44%	↓ Amylase, SOD and CAT activities ↓ Plasma glucose ↑ SGR, WG
Vitamin C	*Leiocassis longirostris*^8^	Ammonia (1.03 and 9.6 mg/L total ammonia nitrogen)	60	0.0038–0.63%	Keeping of serum lysozyme and hepatic SOD activities ↑ SGR, FR
Vitamin E	*Piaractus mesopotamicus*^9^	High stocking density (20 Kg/m^3^)	140	0–0.045%	↓ Plasma cortisol ↑ Kinetic activity of macrophage recruitment ↑ Giant cell formation
Commercial vitamin premix	*Ictalurus punctatus*^10^	Confinement (1 and 6 h)	540	Different mixes	No effect on plasma cortisol
DHA	*Prochilodus lineatus*^11^	Air exposure (60 s)	17	0.13–6.64% TFA (in *Artemia*)	↓ Whole-body cortisol No effect on growth
Astaxanthin	*Pelteobagrus fulvidraco*^12^	Crowding stress (2 days at 150 g/L)	60	0–0.008%g	↓ Serum cortisol and glucose ↓ ALT, AST, ALP and MDA activities ↓ Serum lysozyme activity ↓ SGR, WG
Zn	*Pangasius hypophthalmus*^13^	High lead (Pb) concentration (4 ppm)	75	0–0.002%	↓ Serum cortisol and HSP70 expression ↓ Blood glucose ↑ AChE activity ↓ CAT, SOD, GST, LPO activities

*AchE, Acetylcholine Esterase; ALT, Alanine Aminotransferase; ALP, alkaline phosphatase AST, Aspartate Aminotransferase; CAT, Catalase; FCR, Factor Conversion Rate; FI, Feed Intake; FR, Feeding Rate; GST, Glutathione Transferase HPI, hypothalamus-pituitary-interrenal; LPO, Lipid Peroxydase; MDA, Malondialdehyde;; PER, Protein Efficiency Ratio; SGR, Specific Growth Rate; SOD, Superoxide Dismutase; TFA, Total Fatty Acids; WG, Weight Gain*.

1 Wolkers et al. (48);

2 Morandini et al. (50);

3 Hoseini et al. (59);

4 Martins et al. (58);

5 Abdel-Tawwab (47);

6 Hooley et al. (16);

7 Babaei et al. (12);

8 Liu et al. (20);

9 Belo et al. (63);

10 Davis et al. (72);

11 Araújo and Rosa (79);

12 Liu et al. (24);

13 *Kumar et al. (9)*.

#### Amino Acids

In this group, the works have based on two different biomolecules content in diet: protein/lipid/carbohydrate ratios or tryptophan (Trp), and tilapia (*Oreochromis niloticus*) being the most frequent species. In the former, the study of stress response was a secondary objective beyond the nutritional aspects, meanwhile that response was the main objective in the latter.

Generally, the dietary protein level does not seem to have a significant effect on the stress response in these freshwater species. Concretely, Hooley et al. (16) did not report any plasma cortisol and glucose variations during hauling stress in tilapia; however, these authors pointed that it could be due to a limited ability to detect differences due to the limited number of fish examined at each time point and the high variability in responses between fish within a treatment. Neither Abdel-Tawwab (47) detected differences in plasma cortisol due to overcrowding stress in tilapias fed several protein levels. Lastly, Siberian sturgeon (*Acipenser bareii*) fed different protein, lipids, and carbohydrates levels only showed lower values of cortisol for low carbohydrate diets, regardless protein levels (12).

The effects of Trp-enriched diets on stress and other physiological parameters have been studied in freshwater species. Interestingly, three species have showed a similar stress response, presenting lower cortisol levels in Trp treatments for non-stressed fish, and no variation between those treatments when comparing pre- and post-stress cortisol. Concretely, *Brycon amazonicus* fed Trp supplements reduced their aggressiveness though the plasma cortisol did not vary (48). Contrarily, Martins et al. (58) found differences in plasma cortisol for undisturbed tilapias fed Trp supplements although, curiously, it increased significantly after stress for all treatments (control and Trp added). These authors indicate that despite altering the serotonergic activity, Trp-enriched diets do not always affect the HPI reactivity, as reported by Wolkers et al. (48). Despite Hoseini et al. (59) reported similar responses in Persian sturgeon (*A. persicus*), they went deeper in the study of the endocrine stress response and assessed the variations of serum thyroid hormones. In this sense, these authors have stated that exogenous tryptophan decreases serum levels of thyroid hormones probably via increase in serotonergic activity and elevated cortisol levels.

Only Morandini et al. (50) have reported a post-stress cortisol decrease in chanchita (*Cichlasoma dimerus*) fed Trp supplements. They also described an enhancement of the serotonergic activity hence it seems to be a common physiological reaction derived from this type of diets in studied freshwater species (see above). Those authors also analyzed the plasma sex steroid variations depending on the diet and did not find any differences in those hormones.

#### Vitamins

Commercial vitamin premix did not seem to affect the stress response (cortisol levels) in the Channel catfish (*Ictalurus punctatus*) (72). However, Belo et al. (63) reported that plasma cortisol did not vary in pacu (*Piaractus mesopotamicus*) submitted to overcrowding stress when fed vitamin E supplement (450 mg/Kg). These authors concluded that Vitamin E would seem to act on the stress response of pacus by preventing a stress-related immunosuppression. Contrarily, the serum cortisol levels in *Leiocassis longirostris* submitted to ammonia stress were not affected by the vitamin C supplements, and it was reported that chronic high-ammonia stress showed a tendency to inhibit the cortisol response (20).

#### Lipids and Fatty Acids

In this topic, Araújo and Rosa (79) researched on the effects of the docosaheanoic acid (DHA) in the feeding of *Prochilodus lineatus* larvae. The supplements were provided to the live prey (*Artemia*) during 16 h prior feeding. They stated that supplementation of DHA-rich live feed to *P. lineatus* larvae can attenuate cortisol response to an acute stressor such as air exposure during metamorphosis, when higher mortalities are expected, and the physiological mechanisms underlying the effect of DHA on the larval stress response still need to be elucidated.

Finally, astaxanthin has also been used to reduce stress in yellow catfish (*Pelteobagrus fulvidraco*), stating that this supplement (80 mg/Kg) can improve the anti-oxidative capabilities, hepatic HSP70 levels, and acute overcrowding stress resistance of yellow catfish (24).

#### Minerals

Kumar et al. (9) performed a comprehensive work on the effects of zinc (Zn) supplements on several stressors in the catfish (*Pangasius hypophthalmus*). They studied the integrative stress response to high lead (Pb) concentration, assessing immune, endocrine, metabolic, and oxidative stress parameters. Both plasma stress markers (cortisol and glucose) and oxidative stress enzyme activities improved in fish fed Zn supplements. In addition, immune parameters were enhanced and survival was higher in the experimental diets. Concluding, Zn supplements (10–20 mg/Kg) improved the integrative stress response (endocrine and oxidative) to lead toxicity.

## Conclusions

Overall, the possibility of mitigating the negative effects of stress and disease susceptibility of fish through dietary additives supplementation seems realistic, in particular concerning functional amino acids, fatty acids and minerals. Nevertheless, these nutritional strategies need to take into account several extrinsic (e.g., rearing systems, temperature, salinity, etc.) and intrinsic (e.g., age, genetic background, etc.) factors which in some cases could require tailor-made formulations. The link among the catabolism of those biomolecules and the HPI axis still remains unclear. For instance, the mechanism which serotonin coming from Trp supplements interact with the cortisol/corticosteroid secretion is poorly known.

Further studies are required for validating this nutritional strategy in order to improve welfare and survival in chronically stressed fish. It was observed that both stress response and immune function vary with type of stressors and stress duration. Therefore, once an optimal level of supplementation is achieved for a certain nutrient/additive and for a given species, its beneficial effects should be validated during different stressful conditions commonly found in aquaculture.

## Author Contributions

MH has coordinated the making of the manuscript, collected the most of papers for reviewing, and been the responsible for five main sections. JM has been responsible for three main sections. BC has been responsible for four main sections. All the authors have participated in the final revision of the manuscript.

### Conflict of Interest Statement

The authors declare that the research was conducted in the absence of any commercial or financial relationships that could be construed as a potential conflict of interest.
